# Factors Predicting the Reversal of Hartmann's Procedure

**DOI:** 10.1155/2022/7831498

**Published:** 2022-07-04

**Authors:** Ömer Yalkın, Fatih Altıntoprak, Mustafa Yener Uzunoğlu, Yasin Alper Yıldız, Muhammet Burak Kamburoğlu, Necattin Fırat, Fehmi Çelebi

**Affiliations:** ^1^Department of Surgical Oncology, Bursa City Hospital, Bursa, Turkey; ^2^Department of General Surgery, Sakarya University Training and Research Hospital, Turkey; ^3^Department of General Surgery, Bursa City Hospital, Bursa, Turkey

## Abstract

**Aim:**

This paper investigates the risk factors preventing the reversal and nonreversal of Hartmann's procedure, as a surgical technique that has been performed in our clinic for ten years.

**Methods:**

The study involved a ten-year Hartmann's procedure followed up at our center. The patients were divided into Hartmann reversal and nonreversal groups. Groups were examined in terms of age, gender, diagnosis, stage of malignancy, ASA score, comorbidity, perioperative morbidity-mortality, and the length of the operation.

**Results:**

Age (*p* < 0.001), ASA score (*p* < 0.001), stage in case of malignancy (*p* = 0.002), and comorbidities (*p* < 0.001) were significant risk factors. The ratio of patients without any comorbidities to those with one or more comorbidities was 2.63 (95% CI 1.12–6.20). Among the malignant patients, the ratio of early-stage patients to advanced-stage patients in the group with reversal of Hartmann's colostomy was 2.82 (95% CI 1.30–6.10). In addition, the ratio of older patients to younger patients in group 2 was 0.95 (95% CI 0.92–0.98). A univariate analysis revealed that younger patients, those with lower ASA scores, those without comorbidities, and those with early-stage malignancy had a greater chance of closure of the stoma.

**Conclusion:**

Although Hartmann's procedure is performed in emergency surgery, the nonreversal of the colostomy is a problem in itself. It should be kept in mind that patients who have high risks are likely to have a permanent stoma.

## 1. Introduction

Stoma means “mouth” or “opening” in Greek and was first used as a medical term by French surgeon Pillore in 1774 [1]. In 1921, Henri Hartmann described a stoma procedure that became known as Hartmann's procedure (HP) that is performed especially after the resection of left colon tumors [2]. Hartmann's procedure involves the resection of the unhealthy colonic (left-sigmoid) segment and the diversion of the proximal colon to the end colostomy and the closure of the rectal stump. It is a life-saving procedure in emergencies such as ileus due to rectosigmoid tumors, complicated diverticulitis, gunshot wounds to the colon, inflammatory colitis, volvulus, and primary colonic anastomotic leak/separation [3, 4]. The technique can be applied as a salvage option, benefitting the patient in terms of preventing abdominal fecal peritonitis, which has a mortality rate of up to 30% [5]. Although Hartmann's procedure (HP) is a safe haven in emergencies, colostomy closure operations are associated with high rates of complications, morbidity, and mortality [4, 6]. Hartmann's colostomy has a nonreversal rate ranging from 23% to 74% [7–9]. An alternative approach may include primary anastomosis plus a diverting ostomy [10].

The nonreversal of Hartmann's colostomy is a problem in itself, and the present study investigates the factors predicting and/or affecting the nonreversal of Hartmann's procedure, with the intention being to raise awareness among physicians and patients of the low rate of Hartmann's reversal procedures in high-risk patients.

## 2. Materials and Methods

The study involved a retrospective review of patients who underwent Hartmann's procedure and were followed up at our center between 2008 and 2017. The consent for retrospective clinical study was received from the Clinical Research Ethics Committee. The study included patients over the age of 18 years who underwent HP in our hospital for any reason and who then proceeded or did not proceed to reversal. Patients who died perioperatively after HP, patients under 18 years of age, and patients who were followed up by our center were excluded from the study. In addition, those who underwent HP in another hospital but had colostomy closure in our hospital were also excluded from the study.

Patients who underwent a reversal of Hartmann's colostomy were classified as group 1, while those who did not undergo a reversal were classified as group 2. The gender, age, indications for HP, clinical stage in malignant patients, ASA (American Society of Anesthesiologists) score, comorbidities, postoperative morbidity (Clavien-Dindo classification [11]) and postoperative mortality (for colostomy and reversal operations), length of surgery, and time to reversal of both groups were analyzed.

This study was approved by the ethics committee (informed blinded for peer review).

### 2.1. Statistical Analysis

Statistical data analysis was conducted using IBM SPSS Statistics (version 22.0. Armonk, New York, IBM Corp.). The median values for the variables without normal distribution were presented as median (min–max). For the analysis of quantitative data, Student's *t*-test and Mann-Whitney *U* test were used to determine the differences between means; for the analysis of nonparametric data, a chi-square test was used for the statistical analysis of the associations between groups. A logistic regression analysis was conducted to determine the risk factors affecting Hartman's reversal that was examined in the group with whichever level the *p* < 0.517. The results were considered statistically significant when *p* < 0.05. Odds ratios were calculated, and the confidence intervals of risk levels were presented.

## 3. Results

Among the 303 patients who underwent Hartmann's procedure under emergency conditions between 2008 and 2017, 249 (82.1%) met the inclusion criteria of the present study. Excluded from the study were 32 (10.6%) patients who died preoperatively after HP, eight (2.6%) who were under the age of 18 years, and 14 (4.7%) to whom our center was no longer following up. The patients who underwent HP and colostomy reversal were classified as group 1, while the 149 patients who underwent HP but did not undergo colostomy reversal were classified as group 2. The demographic characteristics of the patients and differences between the groups (*p* values) are presented in Table 1.

Hartmann's colostomy could not be reversed in 59.8% of the patients.

When the patients were evaluated based on their diagnosis, the diagnosis was tumor in 51 (51%), inflammatory causes in 14 (14%), trauma in 13 (13%), diverticulitis in 13 (13%), anastomotic leak in five (5%), and volvulus in four (4%) patients in group 1. In the nonreversal group, in turn, the diagnosis was tumor in 86 (57.7%), inflammatory causes in 16 (10.7%), trauma in seven (4.7%), diverticulitis in seven (4.7%), anastomotic leak in 19 (12.8%), and volvulus in 14 (9.4%) patients (Table 2).

In group 1, 66 of the 100 patients (66%) were male, while in group 2, 95 (63.8%) of the 149 patients were male. There was no statistically significant gender difference between the groups (*p* = 0.717).

The median age of the patients was 57 (18–83) years in group 1 and 70 (22–95) years in group 2. Younger patients were found to be more advantageous in terms of HP reversal (*p* < 0.001).

In groups 1 and 2, 33% and 61.7% of the patients were over 65 years of age, respectively. The number of patients over 65 years of age was lower in group 1 than in group 2 (*p* < 0.001). Being under the age of 65 was more advantageous in terms of HP reversal (*p* < 0.001).

There were 137 malignant and 112 benign patients in total in both groups, with 51 (37.2%) of the malignant patients and 49 (43.8%) of the benign patients being in the group with stoma closure. A comparison of the groups revealed no statistical difference in the rate of reversal between those undergoing colostomy due to a diagnosis of malignant and benign conditions (*p* = 0.296).

Among the total 137 patients who underwent primary surgery with a diagnosis of malignancy, there were patients at stages 1–2 (31 (60.8%) in group 1 and 28 (32.6%) in group 2) and patients at stages 3–4 (20 (39.2%) in group 1 and 58 (67.4) patients in group 2). When the groups were compared, the frequency of stage 3–4 cancer was higher in the nonreversal group (*p* = 0.002). Patients with early-stage malignancy were more advantageous in terms of HP reversal (*p* = 0.002).

There were one or more comorbidities in 35% of the patients (35 patients) in group 1 and 69.8% of the patients (104) in group 2. Patients without comorbidities were more advantageous in terms of HP reversal (*p* < 0.001).

Considering whether multiple comorbidities had an effect on reversal and when the 104 patients in group 2 were evaluated within the group, there were 54 patients with one, 34 patients with two, and 16 patients with three or more comorbidities. A comparison of these subgroups revealed no statistically significant difference (*p* = 0.493).

When the groups were compared in terms of ASA scores, patients with low ASA scores were more advantageous in terms of HP reversal (*p* < 0.001).

When the patients were grouped according to their ASA scores (ASA 1–2 and ASA 3–4), the rate of ASA 3–4 patients was 35% (*n*: 35) in group 1 and 62.4% (*n*: 93) in group 2. Patients with ASA 1–2 were more advantageous in terms of HP reversal (*p* < 0.001).

When all patients were examined in terms of postoperative complications following HP, no complications were identified in 158 (63.5%) patients. Among the 91 patients with complications, 60 (24%) developed surgical site infection, 10 (4%) stomal retraction, 7 (2.7%) enteric fistula, six (2.5%) eventration, six (2.5%) incisional hernia, and two (0.8%) ureteral injury (Table 3).

Morbidity at the time of the first operation was 37% (37) and 34.9% (52) in groups 1 and 2, respectively, with no statistically significant difference between the two groups (*p* = 0.735) in this regard.

When the complications were evaluated using the Clavien-Dindo scoring, there were no complications in 158 (63.5%) of the 249 patients who underwent HP, while 66 patients (26.5%) had Clavien-Dindo grade 1-2-3a and 25 patients (10%) had Clavien-Dindo grade 3b-4a-4b complications. When the groups were compared in this regard, the rate of severe complications (Clavien-Dindo grades 3b-4a-4b) from the first operation in group 1 was 6%, compared with 12.8% in group 2. There was no significant difference between the two groups in this regard (*p* = 0.082).

The median (min–max) length of the first surgery was 150 (45–360) min in group 1, and 140 (45–590) min in group 2, with no statistical difference in this regard (*p* = 0.619).

The median length of the second surgery was 150 (60–430) minutes for the 100 patients in group 1.

The median time to stoma closure was 8 (1–45) months in group 1.

When the perioperative (30-day) morbidity and mortality of the second surgery (Hartmann's reversal) were evaluated, total morbidity was 35% and mortality was 5% for the 100 patients. No complications were observed in 60 (60%) patients. Clavien-Dindo grade 1-2-3a complications were observed in 29 (29%) patients; Clavien-Dindo grade 3b-4a-4b complications in six (6%) patients; and Clavien-Dindo grade 5 complications in five (5%) patients. The complications were surgical site infection in 18 (18%), ileus in seven (7%), anastomotic leak in three (3%), eventration in three (3%), colovesical fistula in one (1%), ureteral injury in one (1%), pneumothorax in one (1%), and pulmonary embolism in one (1%).

A logistic regression analysis was conducted to analyze the multiple risk factors for Hartmann's reversal procedures, and the resulting multiple models were found to be statistically significant (*p* = 0.517).

The ratio of patients without comorbidities to those with comorbidities in the group who underwent Hartmann's reversal procedure was 2.63 (95% CI 1.12–6.20). Among the malignant patients, the ratio of early-stage patients to advanced-stage patients in the group who underwent Hartmann's colostomy reversal was 2.82 (95% CI 1.30–6.10). Furthermore, the ratio of older patients to younger patients in the group who did not undergo Hartmann's procedure reversal was 0.95 (95% CI 0.92–0.98) (Table 4).

A univariate analysis (Table 2) revealed that younger patients, patients with lower ASA scores, those without comorbidities, and those with early-stage malignancy had a greater chance of closure of the stoma. Colostomy and reversal did not pose a significant risk related to any particular gender (*p* = 0.717), pathological diagnosis (*p* = 0.296), complications after the first operation (*p* = 0.735), or length of surgery (*p* = 0.619), while age (*p* < 0.001), ASA score (*p* < 0.001), stage in case of malignancy (*p* = 0.002), and comorbidities (*p* < 0.001) were significant risk factors.

## 4. Discussion

Hartmann's procedure is a life-saving surgical method in emergencies that shortens the length of surgery. It is known that associated morbidities can increase to 50% and mortality to 14% depending on the patient profile, the number of comorbidities, the reason for the operation, and the presence of intra-abdominal sepsis [12, 13].

Previous studies have reported that 6–35% of patients who undergo HP do not proceed to reversal, leading to a permanent stoma [4, 14–17]. Horesh et al. reported Hartmann's reversal rate of 57.6% when those who died within the perioperative 30 days after HP were excluded [18]. In the present study, the rate of Hartmann's reversal was 40.1%, and we discuss here the factors that predict the reversal of HP.

David et al. reported the female gender to be more advantageous in terms of Hartmann's reversal, while in the present study, gender was not found to have a significant effect on HP reversal (*p* = 0.717) [7].

Studies have reported that patients under the age of 70 have a higher chance of colostomy closure and that younger age is an advantage in terms of colostomy closure [3, 19, 20]. Consistent with literature, being under 65 years of age was found to be advantageous in terms of Hartmann reversal in the present study (*p* < 0.001).

Although there have been studies reporting that patients undergoing HP due to a diagnosis of a benign condition have a significantly higher chance of colostomy closure [7, 19, 20], it was observed in the present study that a diagnosis of malignant or benign conditions had no significant effect on colostomy closure (*p* = 0.296). We concluded that this might be due to an excess of patients because of performing HP when primary anastomosis could be performed, and thus, reversal was easier after HP.

It has been reported that patients with low Duke stages had higher rates of Hartmann's reversal [19]. In the present study, early-stage patients (stages 1–2) operated with a diagnosis of malignancy were more advantageous in terms of Hartmann's reversal than advanced-stage patients (*p* = 0.002).

The most important predictive factor for postoperative complications after colostomy closure is multiple medical comorbidities. Almost all reports in the literature indicate that patients with fewer comorbidities have a higher rate of colostomy closure [7, 12, 18, 20]. In the present study, patients with no comorbidities achieved higher rates of Hartmann's reversals than those with one or more comorbidities (*p* < 0.001).

The ASA (American Society of Anesthesiology) score is a significant risk factor for Hartmann's reversal. According to literature, patients with high ASA scores have a low rate of Hartmann's reversal [8, 21–23]. Concurring with literature, it was found in the present study that patients with high ASA scores had a low rate of Hartmann's reversal (*p* < 0.001).

In the present study, the rate of postoperative complications after HP was 37% in group 1 and 34.9% in group 2, which was not significant in terms of Hartmann's reversal (*p* = 0.735). Likewise, when the groups were evaluated in terms of the onset of severe complications (Clavien-Dindo grades 3b, 4a, and 4b), severe complications were recorded in 6% and 12.8% of groups 1 and 2, respectively, which was not significant in terms of Hartmann's reversal (*p* = 0.082). Complications after HP did not affect Hartmann's reversal. While complications after Hartmann's reversal procedures have been reported in literature, there is a lack of data on the effect of complications after Hartmann's procedures on Hartmann's reversal.

Several studies have indicated that the length of surgery is not associated with Hartmann's reversal [22, 24]. In the present study, when the length of HP surgery was evaluated in both groups, it was found to have no effect on Hartmann's reversal (*p* = 0.619).

HP reversal procedures are unpopular among surgeons and are avoided when possible due to the morbidity and mortality rates. Hodgson et al. reported a mortality rate of 4.5% and a morbidity rate of 41% for Hartmann's reversal, while Akıncı et al. [16] reported no mortality and a morbidity rate of 37.5%. In line with literature, in the present study, the morbidity rate of 35% (*n*: 35) and a mortality of 5% (*n*: 5) were identified to be associated with Hartmann's reversal [9, 16]. According to the Clavien-Dindo classification, the rate of grade 1-2-3a and grade 3b-4a-4b complications was 29% and 6%, respectively. When these patients were examined in terms of age, comorbidity, ASA score, morbidity after the first surgery, and diagnosis of malignancy, they were considered to be at high risk of colostomy nonreversal. When the patients who died were examined in terms of age, comorbidity, ASA score, morbidity after the first surgery, and whether there was a diagnosis of malignancy, all five were found to be at high risk for colostomy nonreversal.

The most common complication after Hartmann's reversal is wound site infection, which has been reported to range from 2% to 41% [4]. The presence of wound site infection has been shown to cause wound dehiscence, incisional hernia, prolonged hospital stay, and increased costs [25]. Among our cases, the rate of wound site infection (*n* = 18) following Hartmann's reversal was 18%.

Different opinions have been proposed for the timing of colostomy closure, and so there is still a lack of consensus on this issue. There have been studies suggesting early reversal (45–110 days) [26], while others suggest late reversal (after 6 months) [21]. The mean time to stoma closure was 8 months in the present study.

Tokode et al.'s study of 184 patients included a multivariate analysis investigating the association of colostomy closure with patient age, ASA score, type of admission, presence of extracolonic involvement in cancer cases, Duke classification, and type of pathology [19]. The present study found advanced age, high ASA score, presence of comorbidity, and grade 3–4 malignancies to be negatively associated with colostomy closure. The multivariate analysis conducted in the present study found the ratio of patients without comorbidities to those with comorbidities, the ratio of early-stage patients to advanced-stage patients, and the ratio of older age patients to younger age patients in the reversal Hartmann's colostomy group to be 2.63, 2.82, and 0.95, respectively.

Of the patients in the present study, seven were taken into surgery for the reversal of Hartman's colostomy, but the reversal was unsuccessful due to malignant adhesion. In other words, while all potential risks may be at an acceptable level, Hartmann's colostomy may not be reversed due to technical reasons.

Minimally invasive (laparoscopic, robotic, and single-port) colostomy closures have been associated with less pain, less scarring, earlier mobilization, shorter hospital stays, and less total and surgical morbidity [27]. The most significant problem affecting the operation is the adhesions that may occur as a result of the former operations [4]. In the present series, colostomies were closed laparoscopically in two (2%) cases, while a laparoscopic closure was not possible in three (3%) cases due to malignant adhesions, and the operation was continued using an open surgical technique.

The diverting ileostomy approach following Hartman's colostomy reversal has been used more frequently in recent years for diverticular diseases [28–30]. Prospective multicenter studies have compared primary anastomosis and HP in patients with diverticular peritonitis and have reported that primary anastomosis and ileostomy may be preferred over Hartmann's colostomy. Lee et al. evaluated 2,729 patients who underwent emergency surgery with a diagnosis of diverticulitis and reported that the primary anastomosis with a diverting ileostomy approach did not lead to an increase in mortality or morbidity when compared to HP [10]. Likewise, Arslan et al. indicated that primary anastomosis after resection in the presence of obstructing lesions of the left colon was similar to HP when performed by surgeons with sufficient experience in the aspect of morbidity [24].

## 5. Conclusion

Although Hartmann's procedure is a technique that can be performed in emergencies, the nonreversal of the colostomy is a problem in itself. Our retrospective study found the independent risk factors preventing the reversal of Hartman's colostomy to be advanced age, high ASA score, presence of comorbidity, and advanced-stage disease in cases of malignancy.

It should be understood that the incidence of the reversal of Hartmann's colostomy is low in high-risk patients before making the decision to perform Hartmann's procedure in the first operation. More protective surgical techniques (anastomosis+protective ostomy) should be considered if possible. We recommend that patients be informed in advance that the possibility of stoma closure is low, and that they will continue their lives in this way, with patient and family therapies provided in the early postoperative period.

In addition, it should not be forgotten that the reversal of Hartmann's colostomy is still a major operation in patients at low risk for the nonreversal of Hartmann's colostomy; the total complication rate and morbidity are quite high. It should be known that the rate of postoperative complications is high, especially in cases of multiple comorbidities, and patient selection should be made accordingly. In these patients, more protective surgical techniques should be considered in the initial operation.

## Figures and Tables

**Table 1:** Risk factors for reversal of Hartmann's procedure.

Risk factors	Subgroups	*n*	Group 1	Group 2	*p* value
Gender	Female	88 (35.3%)	34 (34%)	54 (36.2%)	0.717
	Male	161 (64.7%)	66 (66%)	95 (63.8%)	
Age, median (min–max)			57 (18–83%)		<0.001
Age	<65	124 (49.7%)	67 (54%)	57 (46%)	<0.001
	>65	125 (50.3%)	33 (26.4%)	92 (73.6%)	
Pathological diagnosis	Benign	112 (45%)	49 (49%)	63 (42.3%)	0.296
	Malignant	137 (55%)	51 (51%)	86 (57.7%)	
Patients with malignancies	Stages 1–2	59 (43.1%)	31 (60.8%)	28 (32.6%)	0.002
	Stages 3–4	78 (56.9%)	20 (39.2%)	58 (67.4%)	
Comorbidities	Yes	139 (55.8%)	35 (35%)	104 (69.8%)	<0.001
	No	110 (44.2%)	65 (65%)	45 (30.2%)	
ASA	ASA 1	10 (4%)	8 (8%)	2 (1.3%)	<0.001
	ASA 2	111 (44.6%)	57 (57%)	54 (54%)	
	ASA 3	119 (47.8%)	35 (35%)	84 (84%)	
	ASA 1	9 (3.6%)	0 (0%)	9 (9%)	
ASA	ASA 1-2	121 (48.6%)	65 (65%)	56 (37.6%)	<0.001
	ASA 3-4	128 (51.4%)	35 (35%)	93 (62.4%)	
Complications	Yes	89 (35.7%)	37 (37%)	52 (34.9%)	0.735
	No	160 (64.3%)	63 (63%)	97 (65.1%)	
Clavien-Dindo	No-grade 1-2-3a	224 (90%)	94 (94%)	130 (87.2%)	0.082
	Grades 3b-4a-4b	25 (10%)	6 (6%)	19 (12.8%)	
Length of surgery, min, median (min–max)			150 (45–360)	140 (45-590)	0.619

**Table 2:** Diagnoses.

Diagnosis	Group 1	Group 2	*n*
Tumor	51 (51%)	86 (57.7%)	137 (55%)
Trauma	13 (13%)	7 (4.7%)	20 (8%)
Diverticulitis	13 (13%)	7 (4.7%)	20 (8%)
Volvulus	4 (4%)	14 (9.4%)	18 (7.2%)
Other (ischemia, inflammatory causes)	14 (14%)	16 (10.7%)	30 (12%)
Anastomotic leak	5 (5%)	19 (12.8%)	24 (9.6%)

**Table 3:** Complications after Hartmann's Procedure.

Type of complication	First operation (Hartmann's procedure) (*n* = 249)
No complications	158 (63.5%)
Surgical site infection	60 (24%)
Incisional hernia	6 (2.5%)
Stomal retraction or necrosis	10 (4%)
Eventration	6 (2.5%)
Enteric fistula	7 (2.5%)
Ureteral injury	2 (8%)

**Table 4:** Logistic regression.

	Unadjusted		Adjusted	
Risk factors	OR (95% CI)	*p*	OR (95% CI)	*p*
Age	0.95 (0.93–0.97)	<0.001	0.95 (0.92–0.98)	0.002
Stage 1–2	2.91 (1.46–5.80)	0.002	2.82 (1.30–6.10)	0.009
Absence of comorbidities	4.29 (2.50–7.36)	<0.001	2.63 (1.12–6.20)	0.027
ASA 3-4	0.32 (0.19–0.55)	<0.001	0.97 (0.41–2.29)	0.938
Clavien-Dindo 3b-4a-4b^∗^	0.44 (0.17–1.14)	0.089	0.78 (0.16–3.85)	0.757

OR: odds ratio; 95% CI: 95% confidence interval. The *p* value of the Hosmere-Lemeshow test was 0.517; the following factors were included in the multivariate logistic regression analysis: age, grades 1–2, absence of comorbidities, ASA 3–4, and Clavien-Dindo grades 3b-4a-4b. ^∗^Developing a severe complication after the first operation.

## Data Availability

Data used in this study are included in the manuscript.
